# Modulatory Effects of *Lactobacillus paracasei*-Fermented Turmeric on Metabolic Dysregulation and Gut Microbiota in High-Fat Diet-Induced Obesity in Mice

**DOI:** 10.1021/acs.jafc.4c01501

**Published:** 2024-07-04

**Authors:** Wei-Sheng Lin, Siao-En Hwang, Yen-Chun Koh, Pin-Yu Ho, Min-Hsiung Pan

**Affiliations:** † Department of Food Science, National Quemoy University, Quemoy 89250, Taiwan; ‡ Institute of Food Science and Technology, National Taiwan University, Taipei 10617, Taiwan; § Department of Medical Research, China Medical University Hospital, China Medical University, Taichung 40402, Taiwan; ∥ Department of Health and Nutrition Biotechnology, Asia University, Taichung 41354, Taiwan

**Keywords:** obesity, *Lactobacillus paracasei*, turmeric, fermented turmeric, gut microbiota

## Abstract

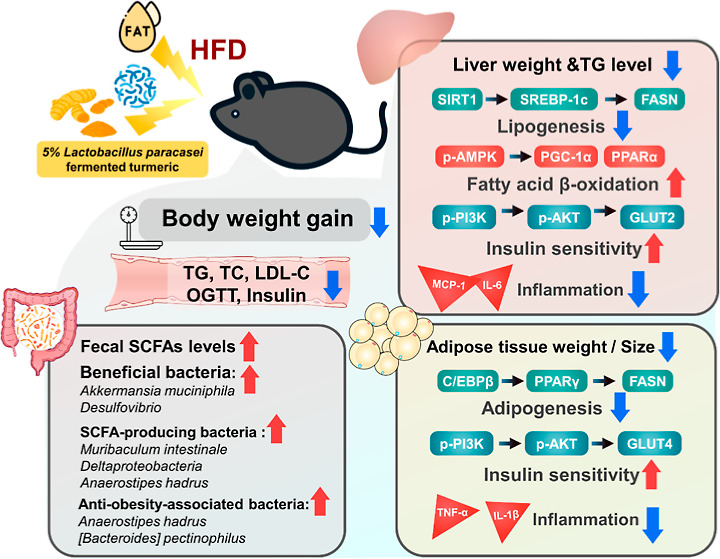

Turmeric, derived from *Curcuma longa*, and *Lactobacillus paracasei*, a lactic acid bacteria, have been studied for their potential antiobesity effects. To date, the antiobesity effects of turmeric fermented with *L. paracasei* have not been sufficiently investigated. This study was conducted *via* oral administration of 5% *L. paracasei-*fermented (FT) and unfermented turmeric (UT) in diet over 16 weeks using high-fat diet (HFD)-induced obese C57BL/6J mice. Results showed that the curcuminoid content of turmeric decreased following fermentation. Furthermore, FT significantly suppressed weight gain and liver and visceral adipose tissue weight and reduced plasma metabolic parameters in both the UT and FT experimental groups. The effects of FT were more noticeable than those of the unfermented form. Moreover, FT downregulated the expression of adipogenesis, lipogenesis, and inflammatory-related protein, but upregulated liver β-oxidation protein SIRT 1, PPARα, and PGC-1α in perigonadal adipose tissue. Additionally, FT ameliorated insulin resistance by activating insulin receptor pathway protein expressions in visceral adipose tissues. FT also modulated gut microbiota composition, particularly in two beneficial bacteria, *Akkermansia muciniphila* and *Desulfovibrio,* as well as two short-chain fatty acid-producing bacteria: *Muribaculum intestinale* and *Deltaproteobacteria*. Our findings indicate that the modulation effect of FT may be an important pathway for its antiobesity mechanisms.

## 1 Introduction

Obesity has emerged as a critical global health issue, with its prevalence nearly tripling since 1975, resulting in various metabolic disorders such as hypertension, diabetes, and nonalcoholic fatty liver disease (NAFLD).^1^ NAFLD, in particular, underscores the critical role of liver metabolism in obesity, characterized by an imbalance between lipid acquisition and disposal, wherein the uptake of fatty acids and de novo lipogenesis exceeds fatty acid oxidation and export. Defined by hepatic steatosis, NAFLD is associated with a myriad of adverse effects and increased mortality.^2^ Considering the negative health impacts and the substantial financial hardship on individuals associated with these conditions, effective dietary interventions are necessary to prevent and mitigate obesity.^3^

Curcumin, a compound derived from *Curcuma longa* L., has been recognized for its potential in obesity management due to its effects on liver health and fat metabolism. Recently, it was reported that *C. longa* oil exhibits hepatoprotective activity and various biological activities, such as antiobesity, antiatherosclerosis, antidiabetes, antimutagenesis, anticancer, and antioxidation effects.^4,5^ Furthermore, curcumin can promote fatty acid oxidation by phosphorylation of ACC and AMPK in 3T3-L1 adipocytes and mouse subcutaneous adipose tissue, reducing HFD-induced weight gain and suppressing angiogenesis.^6^ However, curcumin’s application is limited by its strong taste and low bioavailability.^7^

Recently, plant-based fermentation using probiotic strains is gaining popularity among scientists and the agricultural sector due to its potential to enhance food’s nutritional quality, taste, and smell, as well as to serve as a beneficial supplement for boosting livestock productivity.^8,9^ Furthermore, *Aspergillus oryzae-*fermented products, turmeric, and fermented turmeric (FT) have been shown to demonstrate beneficial effects concerning antiobesity.^10,11^*C. longa* L. fermented with *L. paracasei* has shown promise in reducing scopolamine-induced memory issues, oxidative stress, and inflammation in mice and cell studies.^12^

Preliminary evidence suggests that FT may be more effective in managing obesity than UT, yet comprehensive studies to understand its mechanisms and efficacy are lacking. This study represents an innovative exploration into how fermentation with *L. paracasei* enhances the antiobesity effects of turmeric. While previous research has identified turmeric’s potential in inhibiting fat formation and growth, our work takes a significant step further. Our research hypothesis posits that fermentation of turmeric with *L*. *paracasei* enhances the antiobesity effects of turmeric, leading to improved weight management, metabolic health, and gut microbiota composition. This study specifically examines the potential synergistic effects of natural compounds and fermentation in combating obesity. Using a HFD to induce obesity in C57BL/6J mice, the primary aim of this study is to assess the antiobesity effects of *L. paracasei*-FT. By systematically analyzing the impact of FT on key metabolic pathways, inflammatory responses, and microbiota alterations, we aim to provide comprehensive insights into its mechanisms and potential as a novel dietary strategy against obesity.

## 2 Materials and Methods

### 2.1 Reagents and Antibodies

Antibodies Akt, pAkt (Ser473), AMPK, pAMPK (Thr172), PI3K, and pPI3K were purchased from Cell Signaling Technology (Beverly, MA, USA). FASN, GLUT2, GLUT4, PGC-1α, and Vinculin were purchased from Proteintech (Rosemont, IL, USA). C/EBP β, IL-1β, SIRT 1, SREBP-1c, and TNF-α were purchased from Santa Cruz Biotechnology (Santa Cruz, CA, USA). β-actin, PPAR α, and PPAR γ were purchased from Abcam (Cambridge, UK). The Bio-Rad protein assay dye reagent for protein quantification was obtained from Bio-Rad Laboratories (Munich, Germany), while xylenes, hematoxylin, and eosin for hematoxylin and eosin (H & E) staining were purchased from Leica Biosystems (Nussloch, Germany). Unless specified otherwise, reagents were obtained from Sigma Chemical Co. (St. Louis, MO, USA).

### 2.2 Preparation of UT and FT

Unfermented turmeric (UT) and FT were prepared at Syngen Biotech Co., Ltd. (Taiwan) and stored at 4 °C until use. In the preparation of FT, 2% *L. paracasei* was first inoculated into yeast mold medium and cultured at 37 °C for 6–8 h, after which it was transferred to MRS (deMan, Rogosa, and Sharpe) medium containing turmeric powder to undergo further culturing for 18–20 h. Subsequently, 15% maltodextrin was incorporated as an excipient and the mixture was spray-dried. The use of maltodextrin serves multiple purposes: it acts as a bulking agent to enhance the stability and shelf life of the product, facilitates the spray-drying process by providing the necessary matrix for encapsulation, and improves the palatability and solubility of the final product, which is critical for consistent dosing in experimental designs. Similarly, UT was prepared by spray-drying a mixture of 2% heat-killed *L. paracasei* and 15% excipient maltodextrin, ensuring experimental consistency across test samples.

### 2.3 Sample Composition Analysis

First, 500 μL of a 20 mM ethanol solution of the internal standard methyl red was added to a 0.02 g sample. Then, ethanol was added to bring the volume to 1 mL, the mixture was shaken for 30 min, and the supernatant was collected. Next, 500 μL of acetone (without the internal standard) was added to the remaining solid, and the extraction step was repeated until the precipitate was colorless. Then, the supernatant was collected, dried under nitrogen, redissolved in 1 mL of acetonitrile, filtered through a 0.22 μm nylon filter, subjected to HPLC-UV/vis analysis, and compared with the control group. The area under the curve was used to calculate the curcuminoid content.

### 2.4 Animal Experimental Design and Animal Care

**Figure 1:**
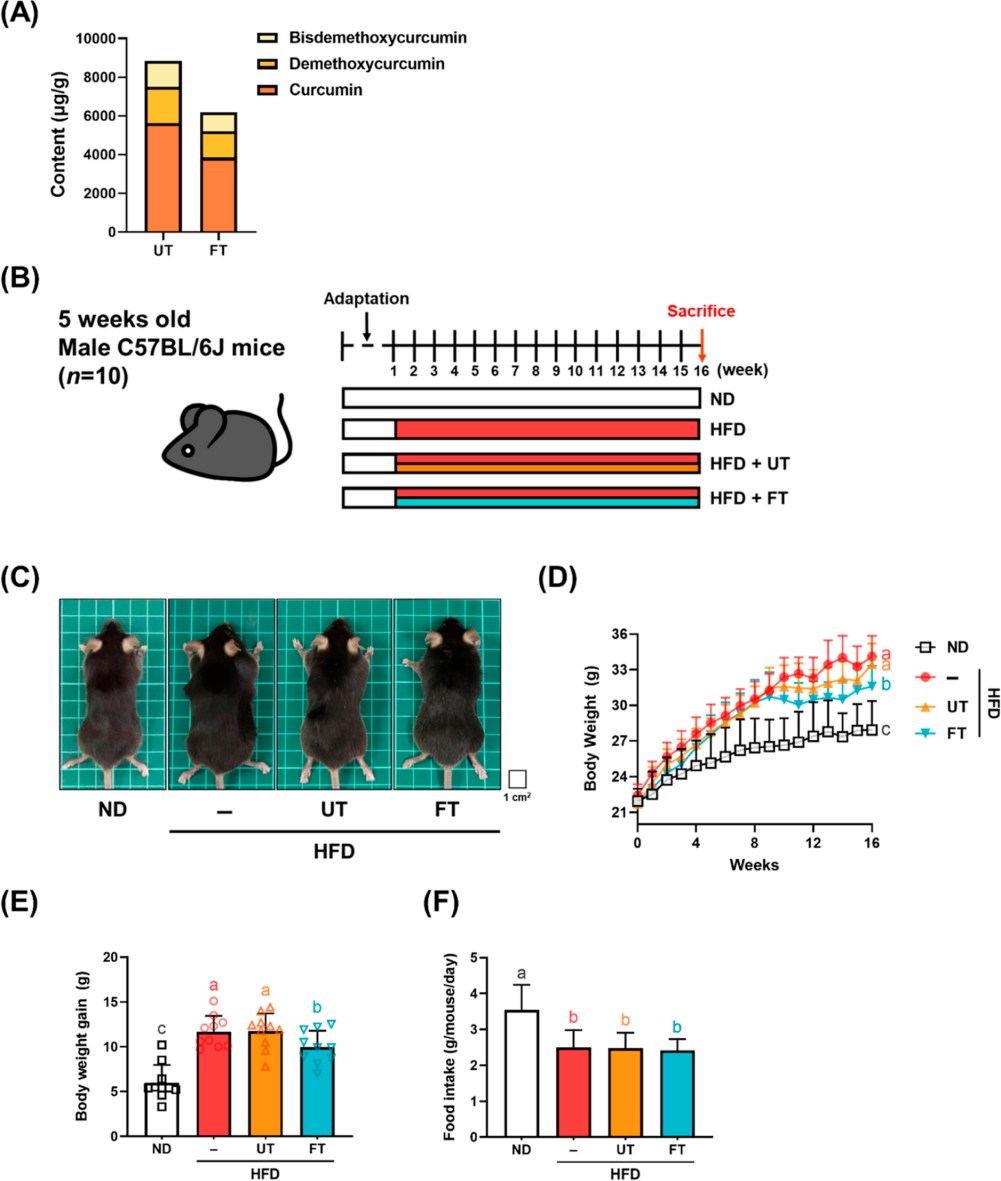
Effect of *L. paracasei*-FT on body weight and food intake in HFD-fed C57BL/6J mice. (A) Curcuminoid contents in UT and FT. The above curcuminoids in UT and FT were measured *via* HPLC. Mice were fed an HFD-supplemented with 5% UT or 5% FT for 16 weeks. (B) Experimental procedure. (C,D) Representative photographs of each group and body weight were recorded weekly. (E) Body weight gain was measured throughout the 16 week period. (F) Food intake. Data are expressed as means ± SD (*n* = 10). Significant difference was analyzed by one-way ANOVA and Duncan’s Multiple Comparison test. Values with different letters (a–c) show significant differences (*p* < 0.05) between each group.

The animal study was designed with eight five-week-old C57BL/6J male mice in each group, for a total of 40 mice purchased from the National Laboratory Animal Center (Taipei, Taiwan). The initial body weights of these mice were closely matched across all experimental groups, ranging from 21.6 ± 0.81 to 22.4 ± 0.92 g, ensuring uniformity at the start of the dietary interventions. The experimental protocols were sanctioned by the Institutional Animal Care and Use Committee of National Taiwan University (NTU-110-EL-00047). Mice were housed under controlled conditions at a temperature of 25 ± 1 °C and relative humidity of 50%, with a 12 h light/dark cycle. Following a week of acclimatization, the mice were systematically allocated to four different diet groups to assess the impact of the fermented and UT on HFD-induced obesity. Animals were given a normal diet (ND, with 13% energy from fat) and a high-fat diet (HFD, 50% energy from fat in diet), and experimental groups were given a HFD with 5% UT and a HFD with 5% FT, respectively. The animal protocol for this study is summarized in Figure 1B. Additionally, the HFD was modified using the Purina 5001 diet (LabDiet, PMI Nutrition International, St Louis, MO, USA), and mice were given free access to food and drinking water. Mice were sacrificed after 16 weeks; all mice were euthanized with CO_2_ and dissected at the end of the study. Blood was immediately collected *via* cardiac puncture and centrifuged to collect serum. The organs, including the liver and visceral adipose tissues, were photographed and weighed. Finally, the serum and organs were frozen at −80 °C before analysis.

### 2.5 Oral Glucose Tolerance Test

Mice underwent oral glucose tolerance test (OGTT) at week 16. After being fasted for 8 h, the glucose level was determined by obtaining blood samples *via* tail nick. The OGTT was performed through fasting blood glucose testing by providing extra glucose at a dose 2 g per kg of body weight *via* oral administration following overnight fasting. Glycemia testing was conducted before providing glucose at the time point of 0 min, followed by time points 30, 60, 90, and 120 min after glucose administration.

### 2.6 Biochemical Analysis

Collected blood samples were centrifuged at 4 °C and 1000*g* for 10 min to separate the serum and blood clots, and blood serums were stored at −80 °C before further analysis. Biochemical analysis was conducted by the National Laboratory Animal Center, NLAC (Taipei, Taiwan). Serums were analyzed for total cholesterol and fasting glucose.

### 2.7 Serum Insulin Level and HOMA-IR

Serum insulin levels were measured using the Mouse Insulin ELISA kit (Mercodia, 10-1247-01) according to the manufacturer’s protocol. At week 16, animals fasted overnight for 8 h, and blood was collected *via* cardiac puncture. The HOMA-IR was calculated using blood glucose (mg/dL) and insulin (mU/mL) levels. HOMA-IR = (fasting glucose × fasting insulin)/405.

### 2.8 Histopathological Examination

Adipose and liver tissues were collected and then fixed in 10% formalin before being dehydrated and paraffinized. Paraffinized tissues were sectioned at 5 μm thickness, followed by deparaffinization and rehydration. Tissue sections embedded in paraffin were stained with H & E for morphological examination. Using ImageJ software, the number and size of adipocytes in subcutaneous adipose tissue were quantified.

### 2.9 Measurement of Triglycerides in the Liver

A sodium phosphate assay buffer and NP 40 substitute assay reagent were used to homogenize the liver, which was then centrifuged at 10,000*g* for 10 min at 4 °C. Lipids were then extracted from the homogenate. To start the reaction, 10 μL of the sample and 150 μL of the diluted enzyme mixture solution were added to each well. The plate was then incubated at room temperature for 60 min. Absorbance was measured at 540 nm using an ELISA reader.

### 2.10 Western Blot Analysis

Liver and adipose tissues were homogenized with ice-cold lysis buffer and placed on ice for at least 1 h. The lysate was then vortexed for a few seconds for a 5 min interval and centrifuged at 12,000*g* for 30 min at 4 °C, and the precipitate was disposed of. Proteins were quantified using a Bio-Rad protein assay before Western blotting analysis. Then, 30 μg of protein samples was loaded into 10% SDS-polyacrylamide gel for electrophoresis, followed by protein transfer onto polyvinylidene difluoride membranes. Transferred membranes were blocked with a blocking solution before overnight incubation with primary antibodies. Incubated membranes were washed with 0.2% phosphate buffer saline Tween 20 three times before and after secondary antibody probing. Protein bands were visualized using chemiluminescence (ECL), and band densities were quantified using Gel-Pro Analyzer software. β-actin and Vinculin were used as internal control for Western blotting.

### 2.11 Next-Generation Sequencing and Gut Microbiota Compositional Analysis

Gut microbiota analysis was performed according to the method described previously.^13^ Genomic DNA from gut microbiota was extracted and purified utilizing a modified InnuPREP Stool DNA kit, followed by forwarding the samples to Biotools Co. Ltd. for fecal microbiome profiling through 16S rRNA amplicon sequencing. The amplification of the 16S rRNA gene encompassed 10 conserved (V3–V4) and 9 hypervariable regions (V1–V9) *via* PCR. Sequencing conducted on the Illumina HiSeq2500 system (250 bp reads) yielded tags, which were subsequently clustered into amplicon sequence variants with a 97% identity threshold, indicative of distinct bacterial taxa at the species or genus level. Microbial data analyses, including α- and β-diversity, were conducted using the Quantitative Insights into Microbial Ecology (QIIME, version 1.9.1) software.

### 2.12 SCFA Analysis

SCFA quantification was conducted as described in a previous study.^14^ This was done by first adding 1 mL of 0.5% phosphoric acid aqueous solution to 0.1 g of feces, homogenizing it for 15 s for two cycles, then adding 0.5 mL of ethyl acetate, homogenizing for 15 s for two cycles, and centrifuging at 4 °C, 18,000*g* for 10 min. Then, 200 μL of the supernatant was collected, and 800 μL of 625 μM internal standard (4-methylvaleric acid) was added to reach a final concentration of 500 μM. Finally, the product was filtered through a 0.22 μm nylon filter for analysis. An Agilent Technologies7890 Gas Chromatograph System with 5975 inert Mass Selective Detector was used to quantify SCFAs in mice feces.

### 2.13 Statistical Analysis

The reported values for the data indicate mean ± standard deviation (SD). At the recognized significance level of *p* < 0.05, a one-way ANOVA followed by a Duncan’s multiple comparison test was used to identify significant group differences. Outliers were defined as data points that were more than two SDs away from the mean. Variations in sample sizes were due to the need for sufficient tissue and serum for each experiment. Each group consisted of at least three specimens.

## 3 Results

### 3.1 Effect of *L. paracasei* Fermentation on Turmeric Powder Curcuminoid Content

Compared with UT, the contents of curcumin, demethoxycurcumin (DMC), and bisdemethoxycurcumin (BDMC) in FT were decreased by 1.46-fold (5627.8 → 3849.2 μg/g), 1.39-fold (1886.4 → 1355.6 μg/g), and 1.38-fold (1340.4 → 970.8 μg/g), respectively (Figure 1A).

### 3.2 Effects of FT on Weight, Food Intake, and Serum Biochemistry in HFD Mice

In HFD-fed mice supplemented with 5% FT for 16 weeks, the increase in body weight was markedly delayed compared to the HFD group (Figure 1C,D). At week 16, the HFD group gained 11.68 ± 0.63 g of weight, which was substantially more than the 9.96 ± 0.58 g gained by the HFD + FT group, suggesting that FT slowed down the HFD-induced weight gain in these mice (Figure 1E). The ND group exhibited higher food intake than the HFD group, which may be attributed to differences in dietary energy density. However, there were no statistically significant differences in food intake among the HFD, UT, and FT groups (Figure 1F). Serum biochemical parameters were assessed to evaluate the effects of administering UT and ferulic acid FT to mice on a HFD. Serum levels of aspartate aminotransferase (AST) and alanine aminotransferase (ALT) were used as indicators of hepatic injury and measured investigate the safety profile of UT and FT interventions. Over a 16 week period, no statistically significant differences in AST and ALT levels were observed among the four experimental groups, suggesting a nontoxic nature of UT and FT regarding their effects on hepatic function. Furthermore, an additional 0.5% cholesterol supplement was added to the HFD regimen to induce dyslipidemia. As shown in Table 1, total cholesterol (TC) and low-density lipoprotein cholesterol (LDL-C) levels were significantly elevated in the HFD group compared to the control group after four months of HFD consumption. Subsequently, both TC and LDL-C showed a declining trend in the UT and FT treatment groups, during which TC concentrations in the UT and FT groups declined by 10.6 and 11.0%, respectively. Concurrently, LDL-C levels showed statistically significant reductions of 3.1 and 3.3% in the UT and FT groups, respectively. Finally, high-density lipoprotein cholesterol (HDL-C) levels in the intervention groups showed no statistically significant differences when compared to the HFD group.

**Table 1:** Effect of *L. paracasei-*FT Supplementation on Serum Biochemical Parameters in HFD-Fed C57BL/6J Mice^a^

item\group	ND	HFD	HFD + UT	HFD + FT
AST (U/L)	87.2 ± 36.5^a^	87.9 ± 39.1^a^	84.68 ± 28.6^a^	84.54 ± 40.1^a^
ALT (U/L)	10.2 ± 1.6^b^	12.3 ± 1.9^a^	13.0 ± 1.4^a^	13.2 ± 2.7^a^
TC (mg/dL)	28.5 ± 3.2^d^	48.1 ± 4.7^a^	43.0 ± 2.5^b^	42.8 ± 4.2^b^
TG (mg/dL)	14.4 ± 2.4^a^	14.5 ± 5.2^a^	3.2 ± 0.9^b^	3.8 ± 1.7^b^
HDL-C (mg/dL)	24.9 ± 3.8^b^	34.5 ± 2.2^a^	34.1 ± 3.2^a^	34.5 ± 3.0^a^
LDL-C (mg/dL)	2.4 ± 0.4^c^	10.0 ± 1.6^a^	8.5 ± 1.2^b^	8.4 ± 0.9^b^

a Data are expressed as mean ± SD (*n* = 8–10 mice/group). Statistical significance of differences among the four groups was analyzed by one-way ANOVA and Duncan’s multiple range tests. Values with different letters (a–c) are significantly different (*p* < 0.05) between each group.

### 3.3 FT Mitigates Hepatic and Visceral Fat Accumulation in HFD Mice

**Figure 2:**
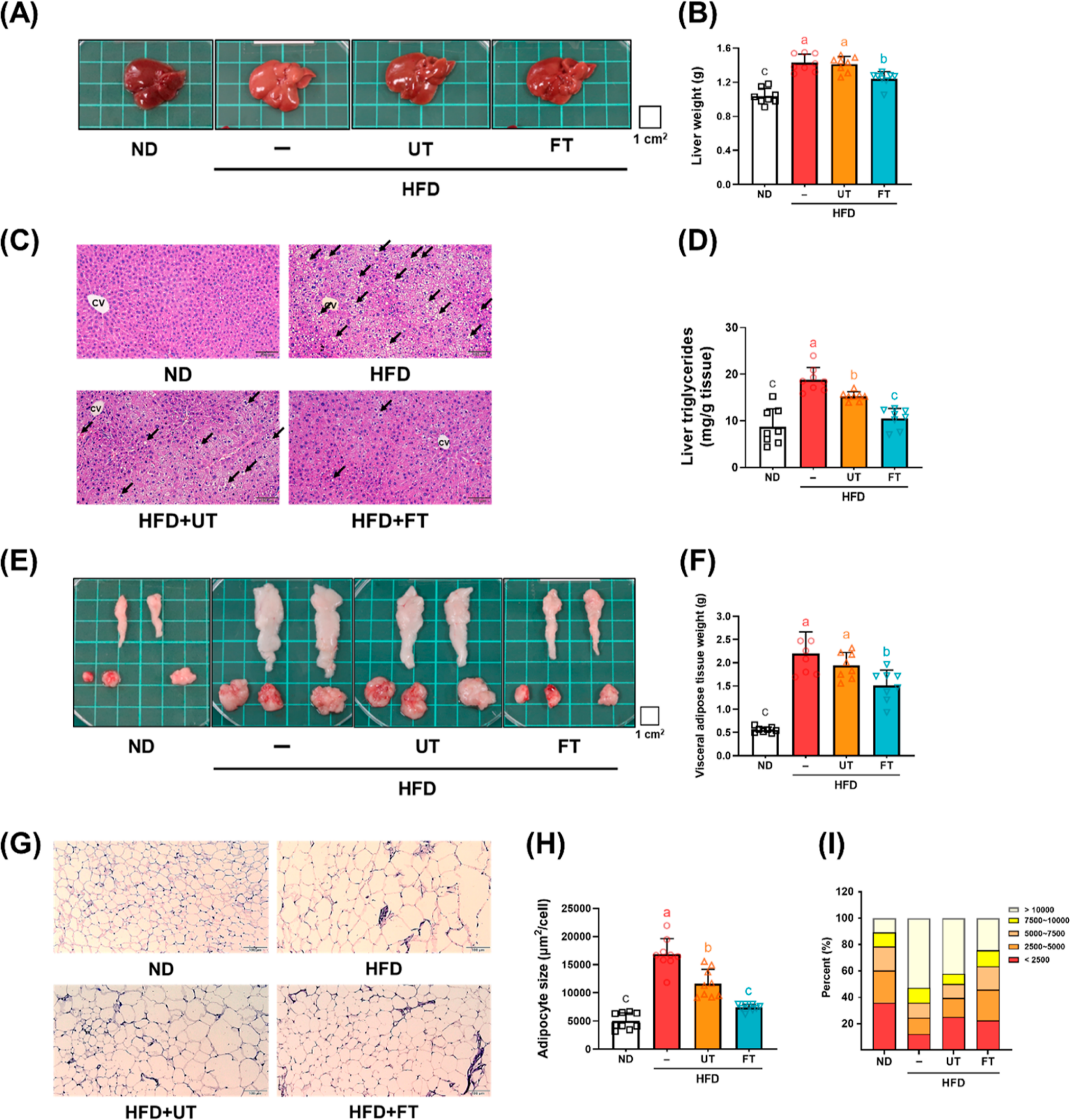
Effect of *L. paracasei*-FT on liver and visceral adipose tissue weight in HFD-fed C57BL/6J mice. (A,B) Representative photographs of each group and corresponding liver weights. (C) 5 μm liver sections were stained with H & E stain (200× magnification). Arrows indicate unstained lipid inclusions. (D) Hepatic TG level. (E) Representative photographs of the visceral adipose tissue (perigonadal, retroperitoneal, and mesenteric fat). (F) Visceral adipose tissue weight. (G) Perigonadal fat was fixed, dehydrated, and embedded; 5 μm adipose sections were stained with H & E stain. Representative photographs of each group (200× magnification). (H,I) Adipocytes’ sizes in visceral adipose tissue were determined by ImageJ. Data are expressed as means ± SD (*n* = 8–10). Significant difference was analyzed by one-way ANOVA and Duncan’s multiple comparison test. Values with different letters (a–c) indicate significant differences (*p* < 0.05) between each group.

Beyond FT’s impact on body weight, its antiobesity properties were also evidenced by reduction hepatic lipid accumulation and visceral adipose tissue size. HFD-fed mice exhibited liver pallor and hepatomegaly, both in appearance and weight. FT supplementation markedly reduced liver weight, indicating an alleviative effect on hepatomegaly (Figure 2A,B). Liver histology (Figure 2C) revealed FT’s role in mitigating micro- and macro-vesicular steatosis, with noticeable decreases in intracytoplasmic fat vacuoles in the HFD + FT group compared to the HFD group. Concurrently, liver triglyceride levels were lower in the UT and FT groups than in the HFD group, underscoring FT’s efficacy in inhibiting hepatic lipid accumulation (Figure 2C,D). Regarding visceral adiposity, HFD-fed mice displayed enlarged perigonadal, retroperitoneal, and mesenteric adipose tissues as compared to ND-fed mice. FT intervention significantly reduced fat storage in these areas. Comparative analysis of each visceral adipose tissue weight is documented in Figure 2E,F, highlighting FT’s comprehensive suppressive effect on visceral adipose tissue growth and resultant lower body fat ratio. Adipocyte size analysis, conducted using ImageJ software and evidenced in H & E stained tissue images (Figure 2H), revealed a predominance of larger adipocytes in the HFD group. In contrast, the UT and FT groups demonstrated moderately sized adipocytes with a higher count under the same magnification. Adipocyte size distribution is quantified in Figure 2I, demonstrating that the percentage of adipocytes measuring 5000–10,000 mm^2^ was significantly higher in the HFD group (52.6%) than in the UT (42.2%) and FT (24.3%) groups. These findings demonstrate that FT effectively counteracted adipocyte enlargement induced by a HFD.

### 3.4 Impact of FT on Adipogenesis and Lipogenesis in Liver and Visceral Fat

**Figure 3:**
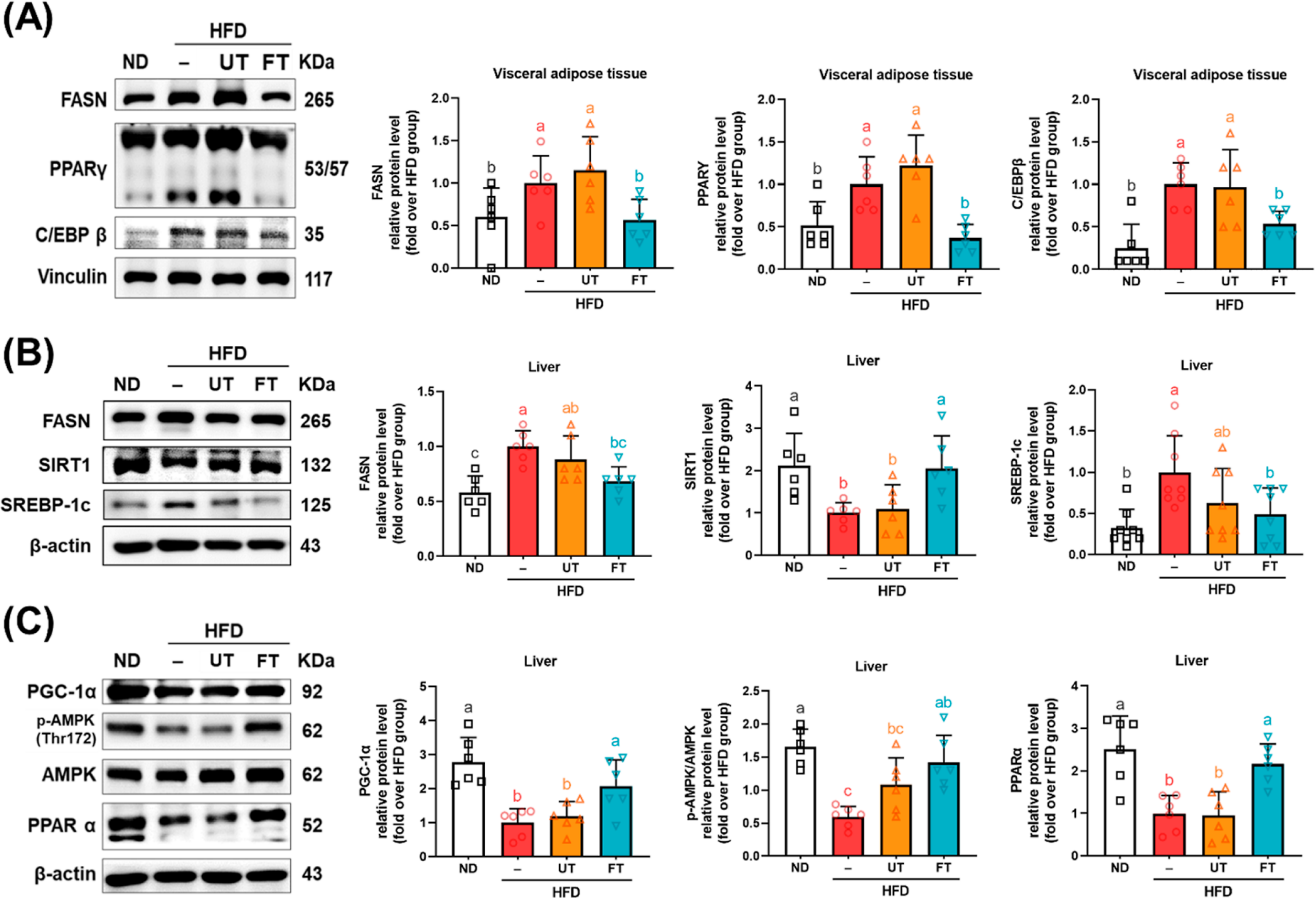
Effect of *L. paracasei*-FT on lipid metabolism-related protein in liver and perigonadal visceral adipose tissue. (A) Relative protein levels of FASN, PPARγ, and C/EBPβ in perigonadal visceral adipose tissue. (B,C) Relative protein levels of FASN, SIRT1, SREBP-1c, PGC-1α, pAMPK, AMPK, and PPARα in the liver were analyzed by Western blotting. β-actin or vinculin was used as the loading control. Data are presented as mean ± SD (*n* = 6). Significant differences were analyzed using one-way ANOVA, followed by a Duncan’s multiple range test. Values with different superscript letters (a–c) indicate significant differences (*p* < 0.05) between groups.

Adipose tissue expansion, characterized by adipocyte hypertrophy, is typically associated with an energy surplus and is mediated through the adipogenesis and lipogenesis processes. In our study, we observed that HFD treatment significantly increased the expression of PPAR-γ, C/EBPβ, and FASN protein levels by 39.8, 48.3, and 75%, respectively, in visceral adipose tissue compared to the ND group. In contrast, no notable differences were detected between the groups fed with UT and the HFD. Interestingly, the expression levels of these proteins were considerably reduced in the group administered with FT as compared to both the HFD and UT groups (*p* < 0.05) (Figure 3A). Hepatic lipid accumulation was governed by a dynamic equilibrium between lipogenesis and fatty acid β-oxidation. In analyzing proteins associated with the lipid synthesis pathway, we found that SIRT1 expression in the HFD group was significantly reduced by 44.5% compared to the ND group. In contrast, the levels of downstream SREBP-1c and FASN were markedly elevated by 64.7 and 37.5%, respectively, implying an enhancement in lipid synthesis due to the HFD. Both UT and FT treatments resulted in increases in SIRT1 expression by 15.4 and 53.9%, respectively, and decreases in SREBP-1c and FASN expressions by 29.5 and 42.3, and 6.2 and 29.2%, respectively. However, significant disparities were observed only between the FT and HFD groups (*p* < 0.05) (Figure 3B). Liver fatty acid β-oxidation, a crucial metabolic pathway, was also investigated here. The HFD group exhibited substantially lower levels of pAMPK/AMPK, reduced by 64%, and its downstream effectors, PPARα and PGC-1α, reduced by 64 and 60.3%, respectively, compared to the ND group. Additionally, the UT group displayed a marginal increase in the pAMPK/AMPK ratio, but no significant changes were noted in PPARα and PGC1-α levels (*p* > 0.05). In contrast, the FT group showed significant elevation in the expression of pAMPK/AMPK, PPARα, and PGC-1α compared to the HFD group (*p* < 0.05) (Figure 3C). This evidence suggests that FT may counteract liver steatosis induced by a HFD.

### 3.5 FT Maintained Blood Glucose Homeostasis in Mice with HFD-Induced Obesity

**Figure 4:**
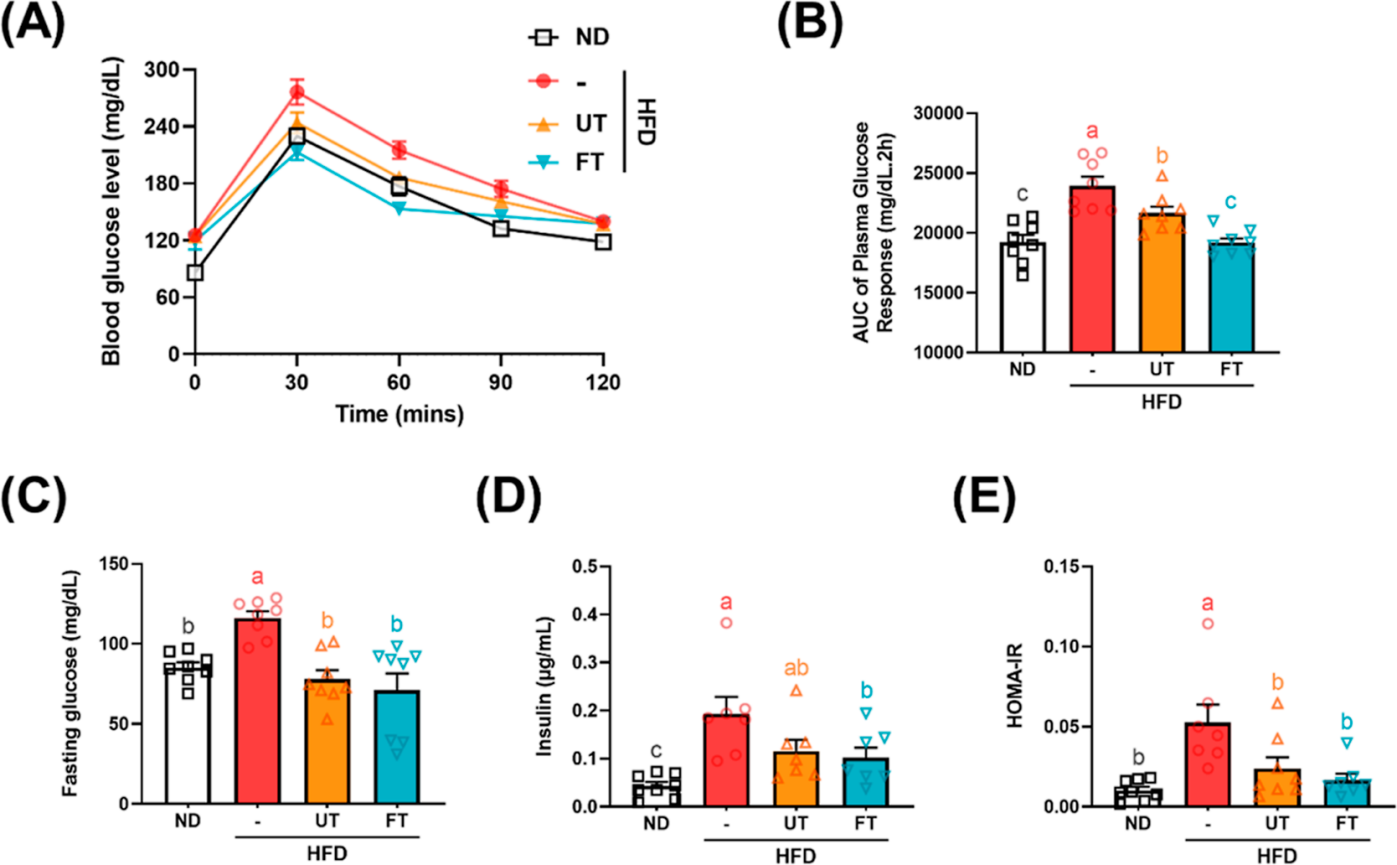
Effect of *L. paracasei*-FT on glucose metabolism in HFD-fed C57BL/6J mice. (A) Glycemic curves during OGTT. (B) Corresponding AUC values. (C) Serum fasting glucose levels. (D) Serum insulin levels. (E) HOMA-IR. Data are presented as mean ± SD (*n* = 8). Significant differences were analyzed using a one-way ANOVA followed by Duncan’s multiple range test. Values with different superscript letters (a–c) indicate significant differences (*p* < 0.05) between groups.

The OGTT serves as a measure of β-cell functionality and ability to modulate serum glucose levels, with lesser fluctuations indicating enhanced glucose tolerance in mice.^15^ As shown in Figure 4A, supplementation with FT in the diet seems to have prevented the elevation of plasma glucose in the OGTT in week 10; this is also significantly supported by the result of AUC. Comparatively, fasting serum glucose levels were significantly elevated in the HFD group relative to the ND group, whereas supplementation with UT and FT resulted in notably lower levels than those observed in the HFD group (*p* < 0.05) (Figure 4C). Insulin concentrations were higher in the HFD group compared to the ND group, with the FT group also exhibiting lower levels than the HFD group (*p* < 0.05) (Figure 4D). The HOMA-IR, an indicator of insulin resistance severity, shows higher values correlating to increased resistance and a propensity for diabetes.^16^ Here, HOMA-IR values were significantly elevated in the HFD group in contrast to the ND group, whereas UT and FT groups showed a reduction in HOMA-IR compared to the HFD group (*p* < 0.05) (Figure 4E).

### 3.6 FT Enhances Insulin Signaling in Liver and Visceral Adipose Tissue

**Figure 5:**
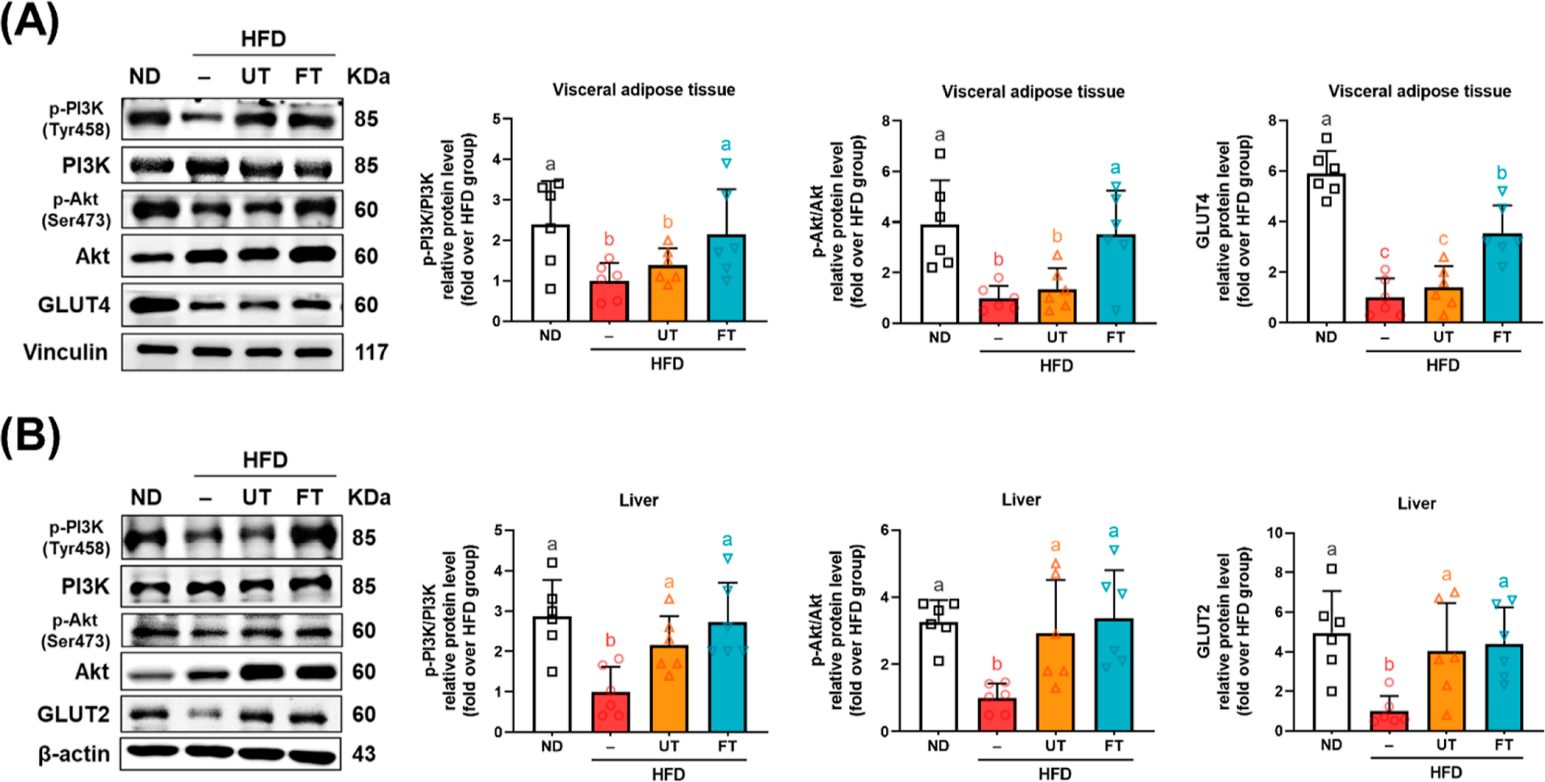
Effect of *L. paracasei*-FT on insulin signaling-related proteins in perigonadal visceral adipose tissue and liver. Relative protein levels of pPI3K, PI3K, pAkt, Akt, GLUT4, and GLUT2 in (A) perigonadal visceral adipose tissue and (B) liver were analyzed by Western blot. Vinculin was used as a loading control. Data are presented as mean ± SD (*n* = 8). Significant differences were analyzed using a one-way ANOVA followed by Duncan’s multiple range test. Values with different superscript letters (a–c) indicate significant differences (*p* < 0.05) between groups.

The activation of the PI3K/Akt signaling cascade, mediated by an array of enzymes, enhances cellular glucose uptake and diminishes extracellular glucose concentrations, thereby effectively mitigating hyperglycemia.^17^ Here, in the perigonadal adipose tissue, a marked diminution was observed in the relative protein levels of pPI3K/PI3K, pAkt/Akt, and GLUT4 by 62.3, 78.1, and 85.7%, respectively, in the HFD group compared to the ND group (*p* < 0.05). Conversely, the UT group exhibited no substantial deviation from the HFD group in terms of pPI3K/PI3K protein levels (*p* > 0.05). Nevertheless, a marginal elevation in downstream pAkt/Akt and GLUT4 levels relative to the HFD group was observed, although these differences did not reach statistical significance (*p* > 0.05). In stark contrast, the FT group demonstrated a significant escalation in the relative protein levels of pPI3K/PI3K, pAkt/Akt, and GLUT4 compared to both the HFD and UT groups (*p* < 0.05), as depicted in Figure 5A. Furthermore, the HFD group exhibited a pronounced reduction in the relative protein levels of pPI3K/PI3K, pAKT/AKT, and GLUT2 in hepatic tissues when contrasted with the ND group (*p* < 0.05). Remarkably, the relative protein levels of pPI3K/PI3K, pAkt/Akt, and GLUT2 in the UT and FT groups were significantly elevated compared to the HFD group (*p* < 0.05), as depicted in Figure 5B.

### 3.7 FT Reduces Inflammation in Liver and Visceral Adipose Tissue

**Figure 6:**
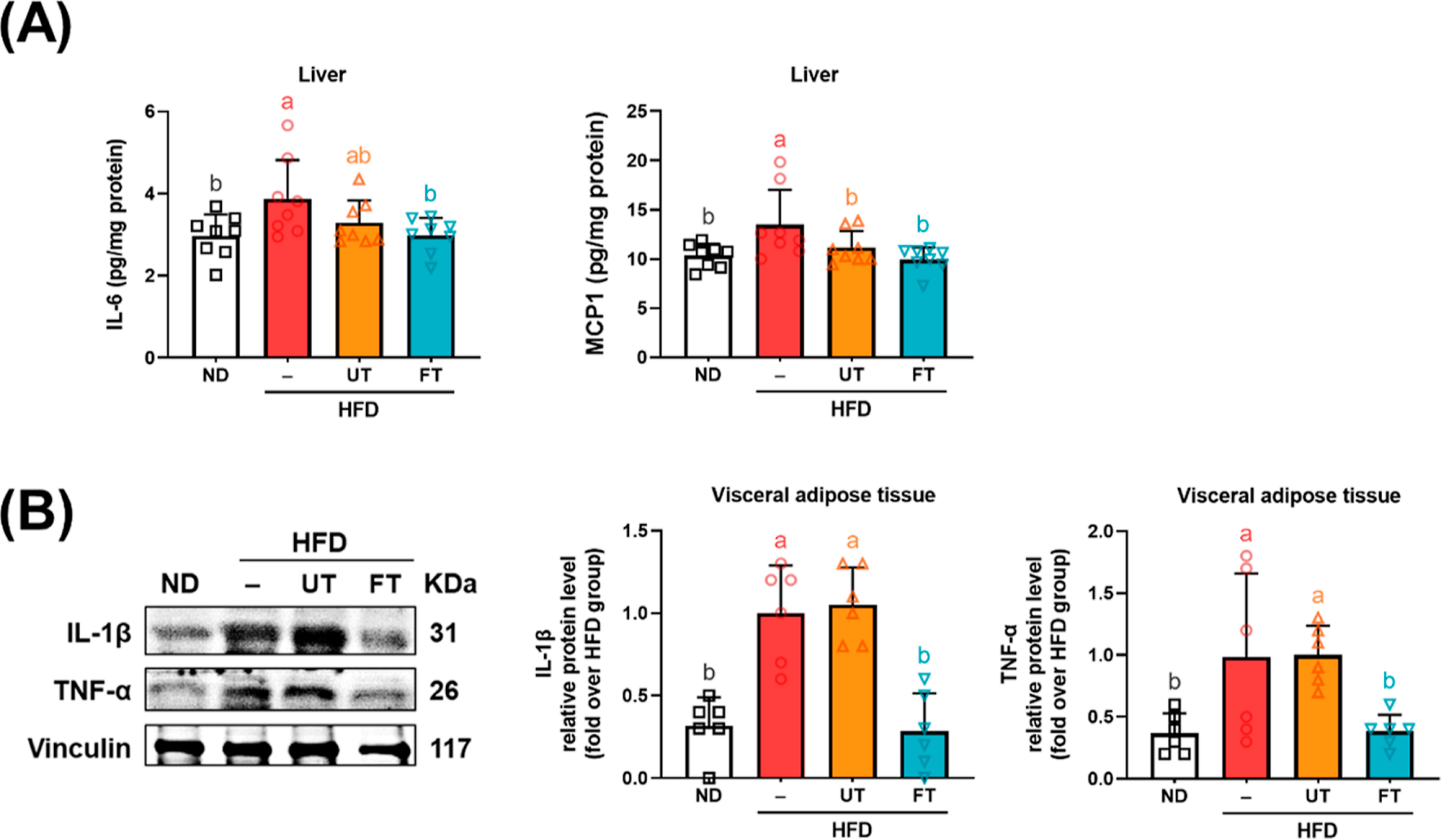
Inhibitory effect of *L. paracasei*-FT on pro-inflammatory responses of HFD-fed mice. (A) IL-6 and MCP-1 levels of liver tissue. (B) Relative protein levels of IL-1β and TNF-α in perigonadal visceral adipose tissue were analyzed by Western blot. Data are presented as mean ± SD (*n* = 6–8). Significant differences were analyzed using one-way ANOVA followed by Duncan’s multiple range test. Values with different superscript letters (a–c) indicate significant differences (*p* < 0.05) between groups.

There is ample scientific evidence indicating a link between obesity and chronic subclinical inflammation.^18,19^ As depicted in Figure 6A, an elevation in hepatic cytokines IL-6 and MCP-1 was observed in the HFD group, with a notable reduction observed in the FT group (*p* < 0.05). Concurrently, the pro-inflammatory cytokines TNF-α and IL-1β in visceral adipose tissue showed significant increases in the HFD group, but were mitigated by FT supplementation (*p* < 0.05) (Figure 6B). These findings suggest that FT has the potential to reduce low-grade inflammation in hepatic and adipose tissues in mice subjected to an HFD.

### 3.8 Effects of FT on Beneficial Bacteria Growth and Fecal Propionic Acid Levels

**Figure 7:**
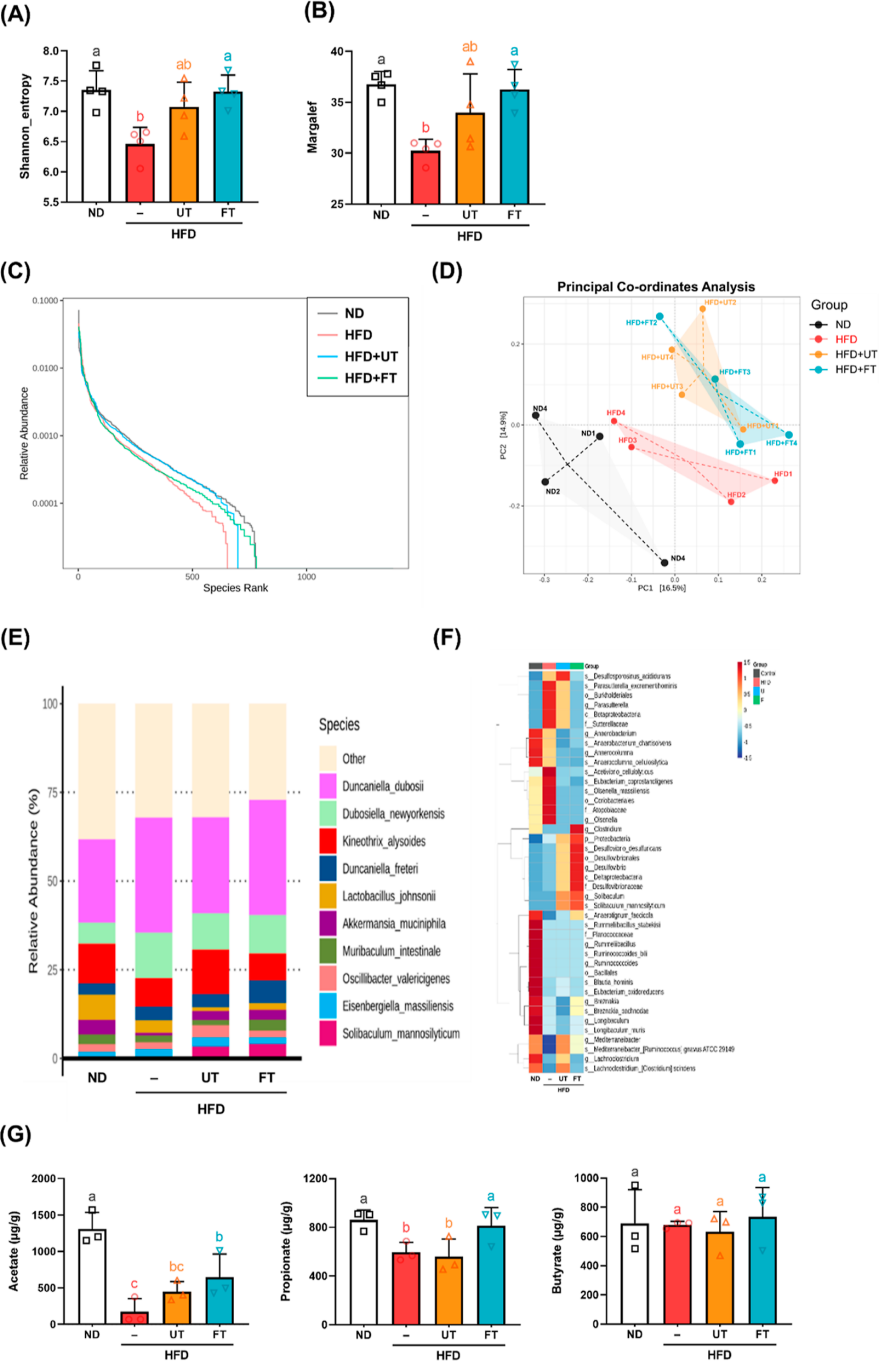
Modulation effects of *L. paracasei*-FT on gut microbiota composition and fecal propionic acid levels in HFD-fed C57BL/6J mice. Gut microbiota composition in feces was analyzed by 16S rRNA gene sequencing analyses (*n* = 4 for each group). (A) Shannon’s diversity index and (B) Margalef’s richness index. (C) Rank abundance curve. (D) Plots shown were generated using principal coordinates analysis (PCoA). (E) Relative abundance of Biomarkers plotted as a clustered heatmap in units of samples and groups. (F) Species-relative abundance of fecal microbiota. (G) Fecal SCFA levels (*n* = 3). Values with different letters (a–b) are significantly different (*p* < 0.05) between each group. Data are presented as mean ± SD. Significant differences were analyzed using one-way ANOVA, followed by a Duncan’s multiple range test. Values with different superscript letters (a–c) indicate significant differences (*p* < 0.05) between groups.

Disturbed gut microbiota, associated with HFD intake, may lead to a reduction in microbiome diversity within the gut.^20^ The concept of alpha diversity serves as a metric for quantifying the diversity of an ecological community. The Shannon indices quantify species evenness, whereas the Margalef indices are used to evaluate species richness. Our findings indicate a significant reduction in these diversity metrics among subjects on a HFD compared to those in the control group (*p* < 0.05). Conversely, after the supplementation with FT, both Shannon and Margalef indices demonstrated an increase compared to the HFD group (*p* < 0.05) (Figure 7A,B).

The rank-abundance curve primarily serves as an illustrative tool to delineate richness in various samples. This curve, characterized by its declining gradient, provides a quantitative measure of species diversity, with a more gradual slope indicating greater diversity. As depicted in Figure 7C, the gut microbiota in the ND and FT groups had elevated species richness relative to the HFD group, which exhibited a diminished bacterial species count. Subsequently, the impact of UT and FT interventions on gut microbiota composition was examined. As shown in Figure 7D, a PCoA revealed that PC1 and PC2 accounted for 16.5 and 14.9% of the variation in intestinal microbial composition, respectively. A notable segregation of the microbial community was evident in the HFD group (central region of the figure) in contrast to the ND group, which is distinct on the figure’s left side. Intriguingly, there is a convergence between the UT and FT groups at the top of the figure. In this study, the HFD group exhibited a lower relative abundance of *Akkermansia muciniphila* than the ND group. Conversely, the UT and FT groups demonstrated increased relative abundance of this species over to the HFD group. Notably, *Desulfovibrio* emerged as the dominant genus in both the UT and FT groups. The administration of FT was also associated with increased presence of short-chain fatty acid-producing bacteria, specifically *Muribaculum intestinale* and *Deltaproteobacteria* (Figure 7E,F). Analysis of SCFAs revealed a pronounced decrease in the fecal concentrations of acetate and propionic acid in the HFD group compared to the ND group. Notably, this decline was reversed following the administration of FT. Conversely, variations in butyrate concentrations did not reach statistically significant differences the groups (Figure 7G).

### 3.9 Gut Microbiome Regulated after FT Supplementation in HFD Mice

**Figure 8:**
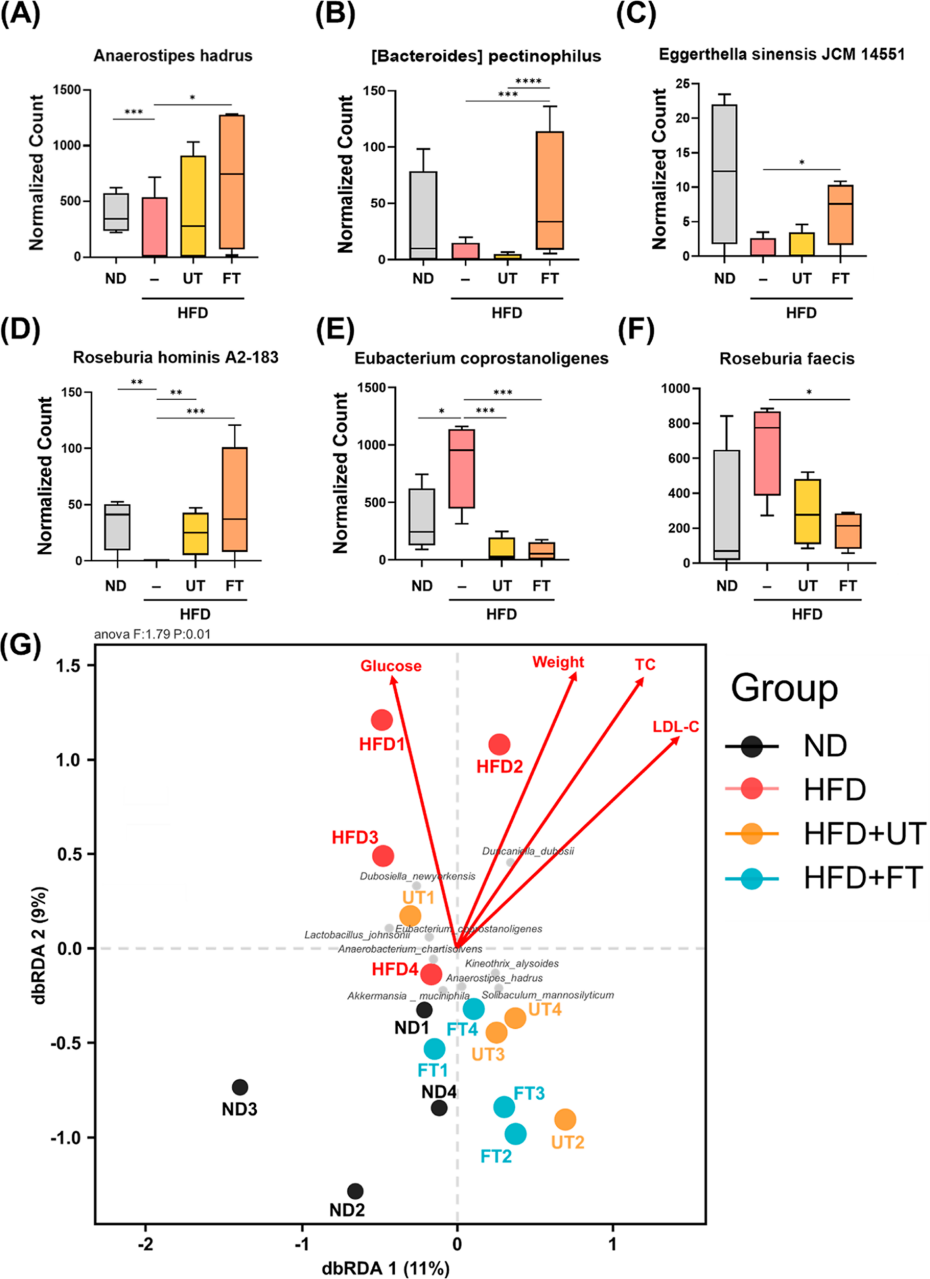
Effects of *L. paracasei*-FT on the abundance of specific gut bacteria and their correlation with obesity parameters in C57BL/6J mice fed a HFD. The alteration of (A) *Eggerthella sinensis* JCM 14551, (B) *Eubacterium coprostanoligenes*, (C) *Roseburia faecis*, (D) *Roseburia hominis* A2-183, (E) *Anaerostipes hadrus*, and (F) [Bacteroides] *pectinophilus*. Abundance of bacteria represented by boxplot. (G) Association between gut microbiota composition and obesity metrics. Distance-based redundancy analysis (db-RDA) quantified species-level correlations. Arrow magnitudes in the schematic reflect the environmental influences on bacterial taxa, while their angular separation delineates correlation types: acute angles signify positive correlations, and obtuse angles, negative correlations. Gray dots denote species, automatically highlighting the top 10 contributors, whereas colored dots signify sample points, categorized by group. Data are presented as means ± SD *p < 0.05; **p < 0.01, ***p < 0.001.

The statistical method of MetagenomeSeq, in the analysis at the species hierarchy level, showed that the FT group increased the abundance of *Anaerostipes hadrus* and [Bacteroides] *pectinophilus* (Figure 8A,B); *Eggerthella sinensis* JCM 14551 was significantly lower in the HFD group than in the ND and FT groups (Figure 8C). Compared to the HFD group, the ND, UT, and FT groups significantly increased the abundance of *Roseburia hominis* A2-183; in contrast, compared to the UT and FT groups, the HFD group significantly increased the abundance of *Eubacterium coprostanoligenes* (Figure 8E). Moreover, compared to the FT group, the HFD group significantly increased the abundance of *Roseburia faecis* (Figure 8F).

To elucidate the complex interrelations among environmental factors, samples, and microbial communities, constrained ordination techniques were employed. Specifically, constrained ordination was utilized to discern the predominant environmental variables that influence the spatial distribution of samples. As depicted in Figure 8G, a dichotomy in correlations with obesity-related factors was observed: the control and FT groups negatively correlated with these factors, implying a protective effect, whereas the HFD group showed a positive correlation, as evidenced by its upward dispersion, suggesting vulnerability to obesity-related influences. Furthermore, the spatial distribution of *A. muciniphila* and *A. hadrus*, especially in the lower quadrant, indicates a diminished correlation with obesity, suggesting a nuanced relationship between these microbial species and obesity-related parameters.

## 4 Discussion

Biotransformation through microbial fermentation enhances the bioavailability of medicinal plants, offering benefits such as specificity, cost-effectiveness, and environmental sustainability.^21^ FT, particularly with *L. paracasei*, has shown significant pharmacological benefits.^11,12^ Turmeric, rich in curcuminoids like curcumin, is noted for its anti-inflammatory and antiobesity properties. Curcumin’s limited bioavailability is attributed to its poor solubility and rapid metabolism, which fermentation can potentially mitigate by altering the curcuminoid structure, thereby enhancing their efficacy.^7,22^ While fermentation decreased the levels of curcumin, DMC, and BDMC (Figure 1A), it may increase the bioactivity of these compounds through microbial transformation, possibly *via* mechanisms such as glycosylation or methylation. It has been suggested that lactic acid bacteria can disaggregate bound phenols in turmeric milk, potentially increasing their bioactivity.^23^ While research shows mixed effects on curcuminoids and total phenolics during fermentation, initial increases followed by decreases have been observed, suggesting dynamic transformations of these compounds.^24,25^ Lim *et al.* noted this pattern during fermentation with *Rhizopus oligosporus*, whereas Xiang *et al.* observed that fermentation with *Monascus purpureus* and *Eurotium cristatum* not only produces new curcuminoids but also alters the balance between major and minor curcuminoids. This complex alteration in phytochemical composition may result from biochemical reactions like glycosylation and methylation, which modify the curcuminoid structure during the fermentation process. Such transformations likely influence the biological activities of fermented products, contributing to their varying effects on health.^26,27^

This study explores the antiobesity mechanisms of *L. paracasei*-FT in HFD-induced obese mice. FT, compared to UT, significantly reduced proteins involved in adipogenesis and lipogenesis in visceral adipose tissue and liver, indicated by the decreased levels of SREBP-1c and FASN and increased SIRT1 expression. Notably, FT enhanced the expression of liver fatty acid β-oxidation proteins, such as pAMPK/AMPK, PPARα, and PGC-1α, suggesting its potential to counter liver steatosis (Figure 3A–C). This process involves the inhibition of key lipogenic proteins by activating AMPKα, which also downregulates adipocyte differentiation transcription factors. The study also highlighted the influence of key active components like Calebin-A, which inhibits lipogenic proteins and fosters phosphorylation pathways leading to metabolic improvements.^28^*L. paracasei* FZU103 was previously found to significantly reduce HFD-induced elevation of *FASN* and *SREBP1* mRNA expression.^29^ Additionally, the activation of AMPK, influenced by components like bisacurone, promotes fatty acid β-oxidation and further supports the metabolic benefits of FT.^30,31^

FT significantly enhanced insulin signaling, as evidenced by the upregulation of PI3K/Akt pathway proteins in adipose and liver tissues, crucial for reducing insulin resistance (Figures 4A–E and 5A,B). Additionally, FT reduced pro-inflammatory cytokines such as IL-6, MCP-1, TNF-α, and IL-1β, which are implicated in obesity-related low-grade inflammation (Figure 6). These findings underscore the potent antiobesity effects of FT, primarily through the modulation of lipid metabolism, inflammation, and insulin sensitivity. The chemokine MCP-1, secreted during lipogenesis by hypertrophic adipocytes, facilitates the differentiation of monocytes into adipose macrophages, promoting macrophage infiltration and the shift from anti-inflammatory M2 to pro-inflammatory M1 macrophages. This shift enhances the secretion of TNF-α and IL-1β, contributing to the inflammatory profile observed in obesity.^32,33^ Concurrently, IL-1β downregulates IRS *via* the extracellular signal-regulated kinase pathway, contributing to insulin resistance.^34^ Moreover, the bioactive component in turmeric, villic acid, potentially restores hepatic IRS, GLUT2, and PI3K, mitigating glucose intolerance and insulin resistance induced by a HFD.^35^

The gut microbiota plays a crucial role in obesity and metabolic health. Notably, compared to UT, FT exhibits a greater potential to modulate the composition of the gut microbiota. During this investigation, it was observed that the group fed an HFD showed reduced species richness in their gut microbiota. However, administering FT in the diet for 16 weeks significantly ameliorated this reduction in species richness in HFD-treated mice (Figure 7A–C). Compared to the ND group, the HFD group in the study exhibited a lower relative abundance of *A. muciniphila*. However, the UT and FT groups demonstrated a higher relative abundance of this species than the HFD group (Figure 7D). The abundance of *A. muciniphila* was negatively correlated with diabetes and obesity. Curcumin’s ability to scavenge oxygen radicals may give bacteria of the *Akkermansia* genus, obligate anaerobic microorganisms, a competitive advantage.^36^ Additionally, lipoteichoic acid from heat-killed *L. paracasei* significantly increased mucin production in enterocytes and enhanced the growth of *A. muciniphila*, which is also a mucin-degrading bacterium.^37^ The research elucidated that the enzymatic degradation of polysaccharides within turmeric powder within the gastrointestinal tract results in the liberation of oligosaccharides. These oligosaccharides are subsequently subjected to microbial fermentation, yielding hydrogen gas as a metabolic byproduct. This cascade of events, in consequence, serves to foster the proliferation of H_2_-utilizing bacterial taxa, notably those belonging to the *Desulfovibrio* genus.^38^ In this experiment, mice fed a HFD with a 5% FT supplement showed a significant increase in the prevalence of short-chain fatty acid-producing bacteria, particularly *Muribaculum intestinale*, *A. hadrus*, and *Bacteroides pectinophilus* (Figures 7D and 8A,B). Compared to the ND group, the HFD group demonstrated a significant decrease in acetate and propionate concentrations, while FT intervention resulted in a significant increase in both these levels. Fermentation products from *L. paracasei* and its exopolysaccharides have been found to stimulate propionate production in both *in vivo* and *in vitro* studies.^39^*A. hadrus* was associated with reduced BMI, body weight, and waist circumference,^40^ while *B. pectinophilus* was more prevalent in nonobese individuals and negatively correlated with blood TG, TC, and LDL.^41^ The abundance of *E. sinensis JCM 14551* is significantly lower in the HFD group compared to the ND and FT groups (Figure 8C). Pinart and colleagues have observed that the genus *Eggerthella* is notably less abundant in obese individuals than in those who are not obese, and it is more common in populations with low inflammation levels.^42^ In contrast, the ND, UT, and FT groups exhibit significantly higher levels of *R. hominis* A2-183 bacteria compared to the HFD group (Figure 8D). Research suggests that this bacterium can boost regulatory T cells, which helps reduce intestinal inflammation.^43^ When compared to the UT and FT, the HFD group shows a significant increase in *E. coprostanoligenes* (Figure 8E), a bacterium whose relative abundance rises in individuals with type 2 diabetes and is directly associated with higher levels of serum TNF-α and TG.^44,45^ Furthermore, the HFD group exhibits a notable increase in *R. faecis* abundance compared to the FT group. This bacterium is more common in obese populations, while the turmeric derivative curcumin β-d-glucuronide has been shown to decrease the abundance of *R. faecis*.^46,47^ A further analysis using constrained ordination was carried out to explore the interactions among environmental factors, samples, and bacterial communities, aiming to pinpoint the environmental factors most significantly influencing the distribution of the samples. As illustrated in Figure 8G, both the ND group and the FT group showed negative correlations with factors related to obesity and positive correlations with the bacteria *A. muciniphila* and *A. hadrus*. Further research, particularly involving human clinical trials, is essential to thoroughly grasp the underlying mechanisms and verify the effectiveness of FT as a potential treatment option for obesity and metabolic disorders.

In summary, our findings suggest that FT may mitigate adipogenesis and lipogenesis in perigonadal white adipose tissue and reduce adipose inflammation, thereby enhancing insulin sensitivity and decreasing lipid accumulation in the liver, with potential benefits against HFD-induced obesity. A few factors limit our study. The specific fermentation process, the concentration of FT used, and how the bioavailability and metabolism of compounds known as curcuminoids change after fermentation might affect the outcomes. Future research should dive deeper into how fermenting turmeric with a particular strain of bacteria, *L. paracasei*, alters the phytochemical makeup of turmeric. Most critically, conducting human clinical trials is crucial to confirm these findings and to determine whether FT can manage obesity and its related metabolic conditions.

## Abbreviations

ACC: acetyl-CoA carboxylase
Akt: protein kinase B
ALT: alanine aminotransferase
AMPK: 5′ adenosine monophosphate-activated protein kinase
AST: aspartate aminotransferase
BMI: body mass index
BSA: bovine serum albumin
C/EBP β: CCAAT/enhancer-binding protein beta
CPT1: carnitine palmitoyltransferase 1
Dlk1: Delta Like Non-Canonical Notch Ligand 1
ECL: enhanced-chemiluminescence
ELISA: enzyme-linked immunosorbent assay
FASN: fatty acid synthase
FT: fermented turmeric
GLUT: glucose transporter
H & E: hematoxylin and eosin
HDL-C: high-density lipoprotein cholesterol
HFD: high-fat diet
HOMA-IR: homeostasis model assessment of insulin resistance
HPLC: high performance liquid chromatography
HRP: horseradish peroxidase
IKKβ: inhibitor of nuclear factor kappa-B kinase subunit beta
IL: Interleukin
INSIG2: insulin induced gene 2
IRS: insulin receptor substrate
JNK: c-Jun N-terminal kinases
LDL-C: low-density lipoprotein cholesterol
MCP-1: monocyte chemoattractant protein-1
NAFLD: nonalcoholic fatty liver disease
OGTT: oral glucose tolerance test
OTUs: operational taxonomic unit
PGC-1α: peroxisome proliferator-activated receptor gamma coactivator 1-alpha
PI3K: phosphoinositide 3-kinase
PPAR: peroxisome proliferator-activated receptor
Pref-1: preadipocyte factor 1
PVDF: polyvinylidene fluoride
SCFAs: short-chain fatty acids
SDS: sodium dodecyl sulfate
SD: standard deviation
SIRT1: sirtuin 1
SREBP-1c: sterol regulatory element binding protein-1c
TC: total cholesterol
TG: triglyceride
TNF-α: tumor necrosis factor-alpha
UT: unfermented turmeric
WHO: World Health Organization.

## References

[ref1] Kloock S.; Ziegler C. G.; Dischinger U. Obesity and its comorbidities, current treatment options and future perspectives: Challenging bariatric surgery?. Pharmacol. Ther. 2023, 251, 108549 10.1016/j.pharmthera.2023.108549. 37879540

[ref2] Tilg H.; Adolph T. E.; Dudek M.; Knolle P. Non-alcoholic fatty liver disease: the interplay between metabolism, microbes and immunity. Nat. Metab. 2021, 3 (12), 1596–1607. 10.1038/s42255-021-00501-9. 34931080

[ref3] Schetz M.; De Jong A.; Deane A. M.; Druml W.; Hemelaar P.; Pelosi P.; Pickkers P.; Reintam-Blaser A.; Roberts J.; Sakr Y.; et al. Obesity in the critically ill: a narrative review. Intensive Care Med. 2019, 45 (6), 757–769. 10.1007/s00134-019-05594-1. 30888440

[ref4] Mun J.; Kim S.; Yoon H. G.; You Y.; Kim O. K.; Choi K. C.; Lee Y. H.; Lee J.; Park J.; Jun W. Water Extract of Curcuma longa L. Ameliorates Non-Alcoholic Fatty Liver Disease. Nutrients 2019, 11 (10), 2536 10.3390/nu11102536. 31640183 PMC6835554

[ref5] Ibáñez M. D.; Blázquez M. A. Curcuma longa L. Rhizome Essential Oil from Extraction to Its Agri-Food Applications. A Review. Plants (Basel) 2020, 10 (1), 44 10.3390/plants10010044. 33379197 PMC7823572

[ref6] Ejaz A.; Wu D.; Kwan P.; Meydani M. Curcumin inhibits adipogenesis in 3T3-L1 adipocytes and angiogenesis and obesity in C57/BL mice. J. Nutr. 2009, 139 (5), 919–925. 10.3945/jn.108.100966. 19297423

[ref7] El-Saadony M. T.; Yang T.; Korma S. A.; Sitohy M.; Abd El-Mageed T. A.; Selim S.; Al Jaouni S. K.; Salem H. M.; Mahmmod Y.; Soliman S. M.; et al. Impacts of turmeric and its principal bioactive curcumin on human health: Pharmaceutical, medicinal, and food applications: A comprehensive review. Front. Nutr. 2023, 9, 1040259 10.3389/fnut.2022.1040259. 36712505 PMC9881416

[ref8] Gupta S.; Abu-Ghannam N. Probiotic fermentation of plant based products: possibilities and opportunities. Crit Rev. Food Sci. Nutr 2012, 52 (2), 183–199. 10.1080/10408398.2010.499779. 22059963

[ref9] Yang X.; Hong J.; Wang L.; Cai C.; Mo H.; Wang J.; Fang X.; Liao Z. Effect of Lactic Acid Bacteria Fermentation on Plant-Based Products. Fermentation 2024, 10 (1), 48 10.3390/fermentation10010048.

[ref10] Lee M.; Nam S. H.; Yoon H. G.; Kim S.; You Y.; Choi K. C.; Lee Y. H.; Lee J.; Park J.; Jun W. Fermented Curcuma longa L. Prevents Alcoholic Fatty Liver Disease in Mice by Regulating CYP2E1, SREBP-1c, and PPAR-α. J. Med. Food 2022, 25 (4), 456–463. 10.1089/jmf.2021.k.0098. 35438556

[ref11] Ho J. N.; Jang J. Y.; Yoon H. G.; Kim Y.; Kim S.; Jun W.; Lee J. Anti-obesity effect of a standardised ethanol extract from Curcuma longa L. fermented with Aspergillus oryzae in ob/ob mice and primary mouse adipocytes. J. Sci. Food Agric. 2012, 92 (9), 1833–1840. 10.1002/jsfa.5592. 22278718

[ref12] Eun C. S.; Lim J. S.; Lee J.; Lee S. P.; Yang S. A. The protective effect of fermented Curcuma longa L. on memory dysfunction in oxidative stress-induced C6 gliomal cells, proinflammatory-activated BV2 microglial cells, and scopolamine-induced amnesia model in mice. BMC Complement. Altern. Med. 2017, 17 (1), 367 10.1186/s12906-017-1880-3. 28716085 PMC5514491

[ref13] Lin W. S.; Chueh T. L.; Nagabhushanam K.; Ho C. T.; Pan M. H. Piceatannol and 3′-Hydroxypterostilbene Alleviate Inflammatory Bowel Disease by Maintaining Intestinal Epithelial Integrity and Regulating Gut Microbiota in Mice. J. Agric. Food Chem. 2023, 71 (4), 1994–2005. 10.1021/acs.jafc.2c08170. 36688924

[ref14] García-Villalba R.; Giménez-Bastida J. A.; García-Conesa M. T.; Tomás-Barberán F. A.; Carlos Espín J.; Larrosa M. Alternative method for gas chromatography-mass spectrometry analysis of short-chain fatty acids in faecal samples. J. Sep. Sci. 2012, 35 (15), 1906–1913. 10.1002/jssc.201101121. 22865755

[ref15] Liu K.-F.; Niu C.-S.; Tsai J.-C.; Yang C.-L.; Peng W.-H.; Niu H.-S. Comparison of area under the curve in various models of diabetic rats receiving chronic medication. Arch. Med. Sci. 2022, 18 (4), 1078–1087. 10.5114/aoms.2019.91471. 35832712 PMC9266878

[ref16] Matthews D. R.; Hosker J. P.; Rudenski A. S.; Naylor B. A.; Treacher D. F.; Turner R. C. Homeostasis model assessment: insulin resistance and beta-cell function from fasting plasma glucose and insulin concentrations in man. Diabetologia 1985, 28 (7), 412–419. 10.1007/BF00280883. 3899825

[ref17] Huang X.; Liu G.; Guo J.; Su Z. The PI3K/AKT pathway in obesity and type 2 diabetes. Int. J. Biol. Sci. 2018, 14 (11), 1483–1496. 10.7150/ijbs.27173. 30263000 PMC6158718

[ref18] McArdle M. A.; Finucane O. M.; Connaughton R. M.; McMorrow A. M.; Roche H. M. Mechanisms of obesity-induced inflammation and insulin resistance: insights into the emerging role of nutritional strategies. Front. Endocrinol. 2013, 4, 52 10.3389/fendo.2013.00052. PMC3650620 23675368

[ref19] Kawai T.; Autieri M. V.; Scalia R. Adipose tissue inflammation and metabolic dysfunction in obesity. Am. J. Physiol. Cell Physiol. 2021, 320 (3), C375–c391. 10.1152/ajpcell.00379.2020. 33356944 PMC8294624

[ref20] Murphy E. A.; Velazquez K. T.; Herbert K. M. Influence of high-fat diet on gut microbiota: a driving force for chronic disease risk. Curr. Opin. Clin. Nutr. Metab. Care 2015, 18 (5), 515–520. 10.1097/MCO.0000000000000209. 26154278 PMC4578152

[ref21] Hegazy M. E.; Mohamed T. A.; ElShamy A. I.; Mohamed A. E.; Mahalel U. A.; Reda E. H.; Shaheen A. M.; Tawfik W. A.; Shahat A. A.; Shams K. A.; et al. Microbial biotransformation as a tool for drug development based on natural products from mevalonic acid pathway: A review. J. Adv. Res. 2015, 6 (1), 17–33. 10.1016/j.jare.2014.11.009. 25685541 PMC4293675

[ref22] Moetlediwa M. T.; Ramashia R.; Pheiffer C.; Titinchi S. J. J.; Mazibuko-Mbeje S. E.; Jack B. U. Therapeutic Effects of Curcumin Derivatives against Obesity and Associated Metabolic Complications: A Review of In Vitro and In Vivo Studies. Int. J. Mol. Sci. 2023, 24 (18), 14366 10.3390/ijms241814366. 37762669 PMC10531575

[ref23] Lu J.-J.; Cheng M.-C.; Khumsupan D.; Hsieh C.-C.; Hsieh C.-W.; Cheng K.-C. Evaluation of Fermented Turmeric Milk by Lactic Acid Bacteria to Prevent UV-Induced Oxidative Stress in Human Fibroblast Cells. Fermentation 2023, 9 (3), 230 10.3390/fermentation9030230.

[ref24] Yong C. C.; Yoon Y.; Yoo H. S.; Oh S. Effect of Lactobacillus Fermentation on the Anti-Inflammatory Potential of Turmeric. J. Microbiol. Biotechnol. 2019, 29 (10), 1561–1569. 10.4014/jmb.1906.06032. 31434176

[ref25] Lo K.-J.; Choudhary S.; Ho C.-T.; Pan M.-H. Exploring the phytochemical composition and pharmacological effects of fermented turmeric using the isolated strain Lactobacillus rhamnosus FN7. J. Food Bioact. 2024, 25, 13 10.31665/jfb.2024.18368.

[ref26] Lim J.; Nguyen T. T. H.; Pal K.; Gil Kang C.; Park C.; Kim S. W.; Kim D. Phytochemical properties and functional characteristics of wild turmeric (Curcuma aromatica) fermented with Rhizopus oligosporus. Food Chem. X 2022, 13, 100198 10.1016/j.fochx.2021.100198. 35499023 PMC9039939

[ref27] Xiang X.; Song C.; Shi Q.; Tian J.; Chen C.; Huang J.; She B.; Zhao X.; Huang R.; Jin S. A novel predict-verify strategy for targeted metabolomics: Comparison of the curcuminoids between crude and fermented turmeric. Food Chem. 2020, 331, 127281 10.1016/j.foodchem.2020.127281. 32559596

[ref28] Lai C. S.; Liao S. N.; Tsai M. L.; Kalyanam N.; Majeed M.; Majeed A.; Ho C. T.; Pan M. H. Calebin-A inhibits adipogenesis and hepatic steatosis in high-fat diet-induced obesity via activation of AMPK signaling. Mol. Nutr. Food Res. 2015, 59 (10), 1883–1895. 10.1002/mnfr.201400809. 26108684

[ref29] Lv X. C.; Chen M.; Huang Z. R.; Guo W. L.; Ai L. Z.; Bai W. D.; Yu X. D.; Liu Y. L.; Rao P. F.; Ni L. Potential mechanisms underlying the ameliorative effect of Lactobacillus paracasei FZU103 on the lipid metabolism in hyperlipidemic mice fed a high-fat diet. Food Res. Int. 2021, 139, 109956 10.1016/j.foodres.2020.109956. 33509508

[ref30] Ashida H.; Tian X.; Kitakaze T.; Yamashita Y. Bisacurone suppresses hepatic lipid accumulation through inhibiting lipogenesis and promoting lipolysis. J. Clin. Biochem. Nutr. 2020, 67 (1), 43–52. 10.3164/jcbn.20-16. 32801468 PMC7417797

[ref31] Smith B. K.; Marcinko K.; Desjardins E. M.; Lally J. S.; Ford R. J.; Steinberg G. R. Treatment of nonalcoholic fatty liver disease: role of AMPK. Am. J. Physiol. Endocrinol. Metab. 2016, 311 (4), E730–e740. 10.1152/ajpendo.00225.2016. 27577854

[ref32] Lumeng C. N.; Bodzin J. L.; Saltiel A. R. Obesity induces a phenotypic switch in adipose tissue macrophage polarization. J. Clin. Invest. 2007, 117 (1), 175–184. 10.1172/JCI29881. 17200717 PMC1716210

[ref33] Kanety H.; Feinstein R.; Papa M. Z.; Hemi R.; Karasik A. Tumor Necrosis Factor α-induced Phosphorylation of Insulin Receptor Substrate-1 (IRS-1). J. Biol. Chem. 1995, 270 (40), 23780–23784. 10.1074/jbc.270.40.23780. 7559552

[ref34] Jager J.; Grémeaux T.; Cormont M.; Le Marchand-Brustel Y.; Tanti J. F. Interleukin-1β-Induced Insulin Resistance in Adipocytes through Down-Regulation of Insulin Receptor Substrate-1 Expression. Endocrinology 2007, 148 (1), 241–251. 10.1210/en.2006-0692. 17038556 PMC1971114

[ref35] Chang W. C.; Wu J. S.; Chen C. W.; Kuo P. L.; Chien H. M.; Wang Y. T.; Shen S. C. Protective Effect of Vanillic Acid against Hyperinsulinemia, Hyperglycemia and Hyperlipidemia via Alleviating Hepatic Insulin Resistance and Inflammation in High-Fat Diet (HFD)-Fed Rats. Nutrients 2015, 7 (12), 9946–9959. 10.3390/nu7125514. 26633482 PMC4690066

[ref36] Guo X.; Xu Y.; Geng R.; Qiu J.; He X. Curcumin Alleviates Dextran Sulfate Sodium-Induced Colitis in Mice Through Regulating Gut Microbiota. Mol. Nutr. Food Res. 2022, 66 (8), e2100943 10.1002/mnfr.202100943. 35106903

[ref37] Wang S.; Ahmadi S.; Nagpal R.; Jain S.; Mishra S. P.; Kavanagh K.; Zhu X.; Wang Z.; McClain D. A.; Kritchevsky S. B.; et al. Lipoteichoic acid from the cell wall of a heat killed Lactobacillus paracasei D3–5 ameliorates aging-related leaky gut, inflammation and improves physical and cognitive functions: from C. elegans to mice. Geroscience 2020, 42 (1), 333–352. 10.1007/s11357-019-00137-4. 31814084 PMC7031475

[ref38] Peterson C. T.; Vaughn A. R.; Sharma V.; Chopra D.; Mills P. J.; Peterson S. N.; Sivamani R. K. Effects of Turmeric and Curcumin Dietary Supplementation on Human Gut Microbiota: A Double-Blind, Randomized, Placebo-Controlled Pilot Study. J. Evid. Based. Integr. Med. 2018, 23, 2515690X1879072 10.1177/2515690x18790725. PMC6083746 30088420

[ref39] Berni Canani R.; De Filippis F.; Nocerino R.; Laiola M.; Paparo L.; Calignano A.; De Caro C.; Coretti L.; Chiariotti L.; Gilbert J. A.; et al. Specific Signatures of the Gut Microbiota and Increased Levels of Butyrate in Children Treated with Fermented Cow’s Milk Containing Heat-Killed Lactobacillus paracasei CBA L74. Appl. Environ. Microbiol. 2017, 83 (19), e01206 10.1128/aem.01206-17. 28733284 PMC5601345

[ref40] Zeevi D.; Korem T.; Godneva A.; Bar N.; Kurilshikov A.; Lotan-Pompan M.; Weinberger A.; Fu J.; Wijmenga C.; Zhernakova A.; et al. Structural variation in the gut microbiome associates with host health. Nature 2019, 568 (7750), 43–48. 10.1038/s41586-019-1065-y. 30918406

[ref41] Brahe L. K.; Le Chatelier E.; Prifti E.; Pons N.; Kennedy S.; Hansen T.; Pedersen O.; Astrup A.; Ehrlich S. D.; Larsen L. H. Specific gut microbiota features and metabolic markers in postmenopausal women with obesity. Nutr. Diabetes 2015, 5 (6), e159 10.1038/nutd.2015.9. 26075636 PMC4491860

[ref42] Pinart M.; Dötsch A.; Schlicht K.; Laudes M.; Bouwman J.; Forslund S. K.; Pischon T.; Nimptsch K. Gut Microbiome Composition in Obese and Non-Obese Persons: A Systematic Review and Meta-Analysis. Nutrients 2021, 14 (1), 12 10.3390/nu14010012. 35010887 PMC8746372

[ref43] Patterson A. M.; Mulder I. E.; Travis A. J.; Lan A.; Cerf-Bensussan N.; Gaboriau-Routhiau V.; Garden K.; Logan E.; Delday M. I.; Coutts A. G. P.; et al. Human Gut Symbiont Roseburia hominis Promotes and Regulates Innate Immunity. Front. Immunol. 2017, 8, 1166 10.3389/fimmu.2017.01166. 29018440 PMC5622956

[ref44] Xiao H. H.; Lu L.; Poon C. C.; Chan C. O.; Wang L. J.; Zhu Y. X.; Zhou L. P.; Cao S.; Yu W. X.; Wong K. Y.; et al. The lignan-rich fraction from Sambucus Williamsii Hance ameliorates dyslipidemia and insulin resistance and modulates gut microbiota composition in ovariectomized rats. Biomed. Pharmacother. 2021, 137, 111372 10.1016/j.biopha.2021.111372. 33761598

[ref45] Ahmad A.; Yang W.; Chen G.; Shafiq M.; Javed S.; Ali Zaidi S. S.; Shahid R.; Liu C.; Bokhari H. Analysis of gut microbiota of obese individuals with type 2 diabetes and healthy individuals. PLoS One 2019, 14 (12), e0226372 10.1371/journal.pone.0226372. 31891582 PMC6938335

[ref46] Khadka S.; Omura S.; Sato F.; Nishio K.; Kakeya H.; Tsunoda I. Curcumin β-D-Glucuronide Modulates an Autoimmune Model of Multiple Sclerosis with Altered Gut Microbiota in the Ileum and Feces. Front. Cell. Infect. Microbiol. 2021, 11, 772962 10.3389/fcimb.2021.772962. 34926318 PMC8677657

[ref47] Andoh A.; Nishida A.; Takahashi K.; Inatomi O.; Imaeda H.; Bamba S.; Kito K.; Sugimoto M.; Kobayashi T. Comparison of the gut microbial community between obese and lean peoples using 16S gene sequencing in a Japanese population. J. Clin. Biochem. Nutr. 2016, 59 (1), 65–70. 10.3164/jcbn.15-152. 27499582 PMC4933688

